# A bound for relative magnetic helicity in terms of free magnetic energy

**DOI:** 10.1007/s11038-025-09578-8

**Published:** 2025-12-08

**Authors:** David MacTaggart

**Affiliations:** School of Mathematics and Statistics, University of Glasgow, Glasgow, G12 8QQ UK

**Keywords:** Magnetic fields, Magnetic helicity, Magnetic topology

## Abstract

The topological complexity of magnetic fields in astrophysics can be described with the aid of magnetic helicity. Amongst its many properties, magnetic helicity provides a bound on the magnetic energy. Whilst this is a very elegant result, classical magnetic helicity, which applies to closed magnetic fields, is not suitable in many astrophysical problems, e.g. modelling active regions in the solar atmosphere. Instead, the classical definition must be replaced by relative helicity, which is able to include open magnetic fields. The purpose of this note is to extend the classical helicity-energy bound to show that an analogous relationship holds between relative magnetic helicity and the free magnetic energy, i.e. the magnetic energy available above the minimum-energy field. The bound is constructed by considering a self-mutual decomposition of relative helicity, which provides information on how the structure of the magnetic field is constrained for a given value of free energy.

## Introduction

Magnetic helicity is a conserved quantity of ideal magnetohydrodynamics (MHD) that encapsulates the topological complexity of a magnetic field. Classical magnetic helicity (hereafter helicity) is defined as

1 $$\begin{aligned} H=\int _\Omega {\varvec{A}}\cdot {\varvec{B}}\,\textrm{d}^3x \quad \textrm{with}\quad {\varvec{B}}\cdot {\varvec{n}}=0\quad \textrm{on}\quad \partial \Omega , \end{aligned}$$

where $\Omega$ is a simply-connected domain with surface unit normal vector ${\varvec{n}}$, ${\varvec{B}}$ is the magnetic field and ${\varvec{A}}$ is a vector potential of the magnetic field, i.e. $\nabla \times {\varvec{A}}= {\varvec{B}}$. In the seminal work of [1] (and later extended in [2]), it was noted that by writing ${\varvec{A}}$ in terms of the Biot-Savart operator, the helicity of linked flux tubes could be interpreted as the Gauss linking number of the closed tubed weighted by the magnetic flux. Whilst such a magnetic setup is highly idealized, the topology-flux interpretation of helicity holds in adaptations suitable for a wide range of applications in astrophysics [3].

As well as its elegant topological interpretation, helicity is also a robust invariant, being approximately conserved in resistive MHD [4] and in the ideal limit of turbulent MHD [5, 6]. This robustness is of fundamental importance in applications, given that several properties of laminar ideal MHD break down in the presence of non-ideal physics and turbulence. Another important physical property of helicity is that it provides a bound for the magnetic energy. Specifically,

2 $$\begin{aligned} |H| \le c_0E_{\varvec{B}}, \end{aligned}$$

where $c_0$ is a constant that depends only on the geometry of the domain [7, 8] and

3 $$\begin{aligned} E_{\varvec{B}}= \frac{1}{2}\int _\Omega |{\varvec{B}}|^2\,\textrm{d}^3x, \end{aligned}$$

is the magnetic energy in $\Omega$. What relation Eq. (2) describes is that as the magnitude of helicity increases, the magnetic energy must also increase. Such an increase may occur in different ways. First, the field strength of ${\varvec{B}}$ may be increased without affecting the topology of the field. Secondly, the topology may be changed so that |*H*| is larger. Thirdly, both these changes may be applied together. The constant $c_0$ depends only on the shape of the domain $\Omega$ [9] and is *independent* of the magnetic field ${\varvec{B}}$. Relation Eq. (2) is a very general statement, but for more specific setups, other topological-based bounds are possible [10].

### Relative helicity

Whilst the understanding of helicity initially developed through its classical definition Eq. (1), this form is not suitable for many astrophysical applications. For example, in understanding the complex magnetic topology in solar active regions, the solar surface (the photosphere) acts as a lower boundary to which the magnetic field connects. Therefore, it is not tangent to the boundary (we refer to such magnetic fields as being *open*, as opposed to *closed* for the classical case) and the classical form *H* is no longer a conserved quantity or gauge-invariant (i.e. independent of the choice of ${\varvec{A}}$). A resolution to this issue was developed by Berger and Field [11] and Finn and Antonsen [12]. In order to develop a form of helicity that can inherit the main properties of classical helicity, two fields with the same boundary conditions are required. That is, for a given magnetic field ${\varvec{B}}\in \Omega$, consider another field ${\varvec{B}}'\in \Omega$ such that ${\varvec{B}}\cdot {\varvec{n}}={\varvec{B}}'\cdot {\varvec{n}}=g(\varvec{x})$ on $\partial \Omega$, for some non-uniform distribution *g* that satisfies

4 $$\begin{aligned} \int _{\partial \Omega }g\,\textrm{d}^2x = 0. \end{aligned}$$

Then, the *relative helicity* is defined as

5 $$\begin{aligned} H_R = \int _\Omega ({\varvec{A}}+{\varvec{A}}')\cdot ({\varvec{B}}-{\varvec{B}}')\,\textrm{d}^3x. \end{aligned}$$

Equation (5) satisfies the gauge-invariance and conservation properties of classical helicity [13, 14]. The reference field ${\varvec{B}}'$ is normally taken to be a potential field. This is because a potential field is, for a given set of boundary conditions, the unique field with the minimum amount of magnetic energy. For the rest of this work, we will take ${\varvec{B}}'$ to be a potential field.

The topological interpretation of relative helicity is different from that of classical helicity. For example, the Gauss linking number is no longer a topological invariant if a flux tube were “cut,” as would be the case for field connected to a boundary. It is beyond the scope of this work to describe how the interpretation of relative helicity changes compared to its classical counterpart, but we note that it is possible to generalize the concept of linking to winding, and we point the interested reader to other works that detail both theory and applications [15–21]. Instead, our focus in the rest of this work will be on how to extend relation Eq. (2) to relative helicity.

## Helicity-energy bound

A first, and naive, approach to finding a suitable bound for relative helicity would be to apply the Cauchy-Schwarz inequality directly to equation Eq. (5). The resulting bound would be

6 $$\begin{aligned} |H_R|= & \left| \int _\Omega ({\varvec{A}}+{\varvec{A}}')\cdot ({\varvec{B}}-{\varvec{B}}')\,\textrm{d}^3x\right| \nonumber \\\le & \int _\Omega |{\varvec{A}}+{\varvec{A}}'||{\varvec{B}}-{\varvec{B}}'|\,\textrm{d}^3x \nonumber \\\le & \left( \int _\Omega |{\varvec{A}}+{\varvec{A}}'|^2\,\textrm{d}^3x\right) ^{1/2} \left( \int _\Omega |{\varvec{B}}-{\varvec{B}}'|^2\,\textrm{d}^3x\right) ^{1/2}\nonumber \\= & 2E_{{\varvec{A}}+{\varvec{A}}'}^{1/2}E_{{\varvec{B}}^*}^{1/2}, \end{aligned}$$

where

$$ E_{{\varvec{C}}} = \frac{1}{2}\int _\Omega |{\varvec{C}}|^2\,\textrm{d}^3x, $$

for ${\varvec{C}}={\varvec{A}}+{\varvec{A}}'$ and ${\varvec{C}}={\varvec{B}}^*:={\varvec{B}}-{\varvec{B}}'$. Note that since we are taking ${\varvec{B}}'$ to be a potential field, the latter integral corresponds to the *free magnetic energy*, i.e. the energy that is available for release from the magnetic field (e.g. via an instability).

The problem with the bound in Eq. (6) is that the coefficient of the (free) magnetic energy depends on the magnetic field through ${\varvec{A}}$, whereas $c_0$ in Eq. (2) is only dependent on the domain geometry. In order to find a better bound for $H_R$ we can make use of a gauge-invariant decomposition [22, 23] of Eq. (5),

7 $$\begin{aligned} H_R= & H_s + H_m \nonumber \\= & \int _\Omega {\varvec{A}}^*\cdot {\varvec{B}}^*\,\textrm{d}^3x + 2\int _\Omega {\varvec{A}}'\cdot {\varvec{B}}^*\,\textrm{d}^3x, \end{aligned}$$

where ${\varvec{A}}^* = {\varvec{A}}-{\varvec{A}}'$ is a vector potential of ${\varvec{B}}^*$. This decomposition can be considered a form of the self-mutual decomposition that is often used for classical helicity [2, 10]. The first term on the right-hand side, $H_s$, represents the *self helicity* and is also referred to as the current-carrying helicity [24]. The second term, $H_m$, represents the *mutual helicity* between the reference field and the current-carrying field. For further details, the reader is directed to [25].

### Bounding $H_s$

Taking the self helicity first, since ${\varvec{B}}^*\cdot {\varvec{n}}=0$ on $\partial \Omega$, this has the form of classical helicity. We can, therefore, without loss of generality, represent the vector potential as

8 $$\begin{aligned} {\varvec{A}}^* = BS({\varvec{B}}^*), \end{aligned}$$

where $BS(\cdot )$ is the Biot-Savart operator, given by

9 $$\begin{aligned} BS({\varvec{B}}^*) = \frac{1}{4\pi }\int _\Omega {\varvec{B}}^*({\varvec{y}})\times \frac{{\varvec{x}}-{\varvec{y}}}{|{\varvec{x}}-{\varvec{y}}|^3}\,\textrm{d}^3y. \end{aligned}$$

The purpose of selecting the Biot-Savart operator is to exploit its self-adjointness. Following Valli [26], the Biot-Savart operator has a complete basis of orthonormal (in $L^2(\Omega )$) eigenfields $\{{\varvec{\omega }}\}_{i=1}^\infty$. The corresponding (real) eigenvalues are denoted $\{\lambda \}_{i=1}^\infty$. We can, therefore, write

$$ {\varvec{B}}^* = \sum _{i=1}^\infty b_i{\varvec{\omega }}_i, \,\,\text{ with }\,\, \Vert {\varvec{B}}^*\Vert ^2_{L^2(\Omega )} = \sum _{i=1}^\infty b_i^2, $$

for $b_i\in \mathbb {R}$, and so,

10 $$\begin{aligned} H_s= & \sum _{i,j}^\infty \int _\Omega b_i{\varvec{\omega }}_i\cdot b_jBS({\varvec{\omega }}_j)\,\textrm{d}^3x \nonumber \\= & \sum _{i,j}^\infty \int _\Omega b_i{\varvec{\omega }}_i\cdot b_j\lambda _j{\varvec{\omega }}_j\,\textrm{d}^3x = \sum _{i=1}^\infty b_i^2\lambda _i. \end{aligned}$$

If we denote $\lambda _\textrm{max}$ to be the eigenvalue with the largest magnitude, then

$$ |H_s| = \left| \sum _{i=1}^\infty b_i^2\lambda _i\right| \le |\lambda _\textrm{max}|\sum _{i=1}^\infty b_i^2 = 2|\lambda _\textrm{max}| E_{{\varvec{B}}^*}. $$

Now if ${\varvec{\omega }}_\textrm{max}$ is the eigenfield associated with $\lambda _\textrm{max}$, then we can write

11 $$\begin{aligned} BS({\varvec{\omega }}_\textrm{max}) = \lambda _\textrm{max}{\varvec{\omega }}_\textrm{max}. \end{aligned}$$

To determine the value of this eigenvalue in practice, it is noted that this is related to the lowest, in magnitude, eigenvalue of the force-free field [27],

12 $$\begin{aligned} \nabla \times {\varvec{\omega }}_\textrm{max} = \frac{1}{\lambda _\textrm{max}}{\varvec{\omega }}_\textrm{max}. \end{aligned}$$

Although analytical results are few (e.g. [27, 28]), $1/\lambda _\textrm{max}$ can be determined numerically for both simply connected [29] and multiply connected [30] domains. It is the shape of the domain that determines the value of $\lambda _\textrm{max}$.

It is clear from the discussion that $c_0=2|\lambda _\textrm{max}|$ as the self-helicity has the form of classical helicity. In this way, the classical bound Eq. (2) will form a special case of the bound for relative helicity.

### Bounding $H_m$

For the mutual helicity, we may now make use of the Cauchy-Schwarz inequality to produce an effective bound,

13 $$\begin{aligned} |H_m|= & 2\left| \int _\Omega {\varvec{A}}'\cdot {\varvec{B}}^*\,\textrm{d}^3x\right| \nonumber \\\le & 2\int _\Omega |{\varvec{A}}'\cdot {\varvec{B}}^*|\,\textrm{d}^3x \nonumber \\\le & 2\left( \int _\Omega |{\varvec{A}}'|^2\,\textrm{d}^3x\right) ^{1/2}\left( \int _\Omega |{\varvec{B}}^*|^2\,\textrm{d}^3x\right) ^{1/2} \nonumber \\= & 4E_{{\varvec{A}}'}^{1/2}E_{{\varvec{B}}^*}^{1/2}. \end{aligned}$$

At first sight, it looks as if we have not made progress compared to the bound in Eq. (6). This is not the case, however, as the coefficient of the free magnetic energy only depends on ${\varvec{A}}'$, the vector potential of the reference field, and not ${\varvec{A}}$. This distinction is important because our aim is to find a bound like Eq. (2) such that the coefficient is not a function of the ${\varvec{B}}$. If we consider a particular domain $\Omega$ with a particular set of boundary conditions ${\varvec{B}}\cdot {\varvec{n}}=g$ on $\partial \Omega$, then we may choose a particular ${\varvec{A}}'$ which will be suitably gauge-invariant for any value of ${\varvec{B}}$. Further, we can choose ${\varvec{A}}'$ to minimize $E_{{\varvec{A}}'}$, thus providing the best bound for Eq. (13).

First, consider the change of gauge

14 $$\begin{aligned} {\varvec{A}}' \rightarrow {\varvec{A}}' + \nabla \phi , \end{aligned}$$

where $\phi$ is a scalar function. The “energy” of this gauge change is

15 $$\begin{aligned} \Vert {\varvec{A}}' + \nabla \phi \Vert _{L^2(\Omega )}^2 =\int _\Omega |{\varvec{A}}'|^2\,\textrm{d}^3x +\int _\Omega |\nabla \phi |^2\,\textrm{d}^3x + 2\int _\Omega {\varvec{A}}'\cdot \nabla \phi \,\textrm{d}^3x \end{aligned}$$

The first two terms on the right-hand side of Eq. (15) are clearly non-negative. The third term can be rewritten using the divergence theorem,

16 $$\begin{aligned} \int _\Omega {\varvec{A}}'\cdot \nabla \phi \,\textrm{d}^3x = \int _{\partial \Omega }\phi {\varvec{A}}'\cdot {\varvec{n}}\,\textrm{d}^2x - \int _\Omega \phi \nabla \cdot {\varvec{A}}'\,\textrm{d}^3x. \end{aligned}$$

Therefore, if we enforce that ${\varvec{A}}'$ satisfies

17 $$\begin{aligned} \nabla \cdot {\varvec{A}}' =0\,\,\textrm{in}\,\,\Omega , \quad {\varvec{A}}'\cdot {\varvec{n}}= 0\,\,\textrm{on}\,\,\partial \Omega , \end{aligned}$$

then the last term in Eq. (15) disappears. The result is that ${\varvec{A}}'$ subject to the conditions in Eq. (17) minimizes $E_\mathrm{{\varvec{A}}'}$. This gauge choice is known as the *Coulomb gauge* and was also used in the bound for $H_s$ (the Biot-Savart operator also satisfies these conditions). The conditions in Eq. (17) appear in diverse areas of physics, particularly in relation to some underlying topological property in a physical system [31]. This gauge has also been used in relative helicity calculations related to force-free extrapolations of solar active regions [32]. The coefficient in the bound in Eq. (13) can, therefore, be calculated routinely.

### A combined bound

Given the results above, we may now write

18 $$\begin{aligned} |H_R| \le c_0E_{{\varvec{B}}^*} + c_1E_{{\varvec{B}}^*}^{1/2}, \end{aligned}$$

where, for *fixed* boundary conditions on a *fixed* domain, the coefficients $c_0$ and $c_1$ are constants defined by

19 $$\begin{aligned} c_0 = 2|\lambda _\textrm{max}|, \quad c_1 = 4E_{{\varvec{A}}'}^{1/2}, \end{aligned}$$

with $${\varvec{A}}'$$ satisfying the conditions in Eq. (17). The bound in Eq. (18) represents a generalization of that in Eq. (2) in the sense that, first, the coefficients only depend on the domain geometry and the *chosen* and $$fixed $$ boundary conditions, and secondly, Eq. (18) reduces to Eq. (2) if the magnetic field becomes everywhere tangent to the boundary (i.e. the magnetic field changes from being open to being closed). Whilst our focus here in on the theoretical result, the bound in Eq. (18) may be determined numerically with existing methods, as cited previously.

Due to the self-mutual decomposition, the bound in Eq. (18) can provide us with some extra information about the relationship between relative helicity and free magnetic energy. For a given value of $E_{{\varvec{B}}^*}$, the maximum possible magnitude of the relative helicity is determined by the bounds on both the mutual and self helicities. There is a special value for which the maximal magnitude of relative helicity is bounded by a balance of the magnitudes of self and mutual components. This specific value corresponds to a free energy

20 $$\begin{aligned} E_{{\varvec{B}}^*}^{0} = \left( \frac{c_1}{c_0}\right) ^2, \end{aligned}$$

and a corresponding maximal relative helicity magnitude

21 $$\begin{aligned} |H_R|_\textrm{max} = \frac{2c_1^2}{c_0}. \end{aligned}$$

**Fig. 1:**
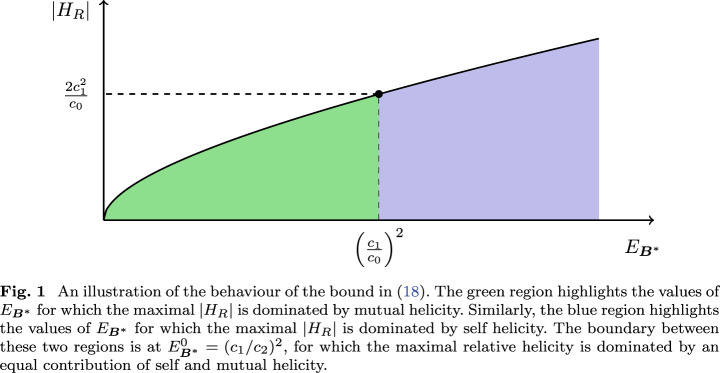
An illustration of the behaviour of the bound in Eq. (18). The green region highlights the values of $E_{{\varvec{B}}^*}$ for which the maximal $|H_R|$ is dominated by mutual helicity. Similarly, the blue region highlights the values of $E_{{\varvec{B}}^*}$ for which the maximal $|H_R|$ is dominated by self helicity. The boundary between these two regions is at $E^0_{{\varvec{B}}^*}=(c_1/c_2)^2$, for which the maximal relative helicity is dominated by an equal contribution of self and mutual helicity

For this particular value of the free energy, the relative helicity magnitude is maximized when both the self and mutual magnitudes are maximized and have the same value. For $E_{{\varvec{B}}^*}<E_{{\varvec{B}}^*}^{0}$ the maximal magnitude of relative helicity is dominated by mutual helicity, whereas for $E_{{\varvec{B}}^*}>E_{{\varvec{B}}^*}^{0}$ it is dominated by self helicity. These properties are illustrated in Fig. 1.

## Summary and discussion

In this note, we have extended the helicity-energy bound, as applied to classical helicity, to relative helicity. In particular, we have derived a bound for the magnitude of relative helicity in terms of a function of the free magnetic energy involving constants that depend on the domain geometry and the normal component distribution of the magnetic field on the domain boundary. Therefore, given a fixed domain and boundary conditions, the bound in Eq. (18) describes how relative helicity can be maximized for a given value of the free magnetic energy.

The bound is split into two parts based on the gauge-invariant decomposition of relative helicity into self and mutual parts. This choice results in a particular value for the free magnetic energy, $E^0_{{\varvec{B}}^*}$, below which the maximal relative helicity magnitude is dominated by the mutual part and above which it is dominated by the self part. Of course, a particular magnetic field may have more self than mutual helicity below $E^0_{{\varvec{B}}^*}$, or more mutual than self helicity above this value, but this means that the relative helicity magnitude is not maximized. The value of the free magnetic energy, for which the maximal relative helicity represents a balance of the contributions from the self and mutual parts, depends on the domain geometry and the boundary conditions, two properties that are independent of the strength of the magnetic field inside the domain.

The bound in Eq. (18) can be used for the analysis of magnetic fields. For example, in an extrapolation of the magnetic field of a solar active region, the bound could help to provide information about the probable dynamics of the region. If the region were such that $E_{{\varvec{B}}^*}<E^0_{{\varvec{B}}^*}$ and possessed a value of $|H_R|$ close to the maximum value, this may indicate that the current configuration of the active region magnetic field is unlikely to produce an eruption (as such eruptions typically involve structures related to self helicity). If, on the other hand, $E_{{\varvec{B}}^*}>E^0_{{\varvec{B}}^*}$ and $|H_R|$ were close to the maximal value, this may indicate that the magnetic field would be highly twisted and unstable. For a particular active region, the value of $E^0_{{\varvec{B}}^*}$ may also be useful as an indicator of when a particular magnetic field may become eruptive, since, as mentioned above, instabilities are typically associated with twisted magnetic field that is described by self helicity.

## Data Availability

Data sharing is not applicable to this article as no datasets were generated or analyzed during the current study.
